# Rosiglitazone ameliorates tissue plasminogen activator‐induced brain hemorrhage after stroke

**DOI:** 10.1111/cns.13260

**Published:** 2019-11-22

**Authors:** Yan Li, Zi‐Yu Zhu, Bing‐Wei Lu, Ting‐Ting Huang, Yue‐Man Zhang, Na‐Ying Zhou, Wei Xuan, Zeng‐Ai Chen, Da‐Xiang Wen, Wei‐Feng Yu, Pei‐Ying Li

**Affiliations:** ^1^ Department of Anesthesiology Renji Hospital School of Medicine Shanghai Jiaotong University Shanghai China; ^2^ Department of Radiology Renji Hospital School of Medicine Shanghai Jiaotong University Shanghai China

**Keywords:** blood‐brain barrier, cerebral ischemia, hemorrhagic transformation, microglia, Rosiglitazone, stroke, tPA

## Abstract

**Objective:**

Delayed thrombolytic therapy with recombinant tissue plasminogen activator (tPA) may exacerbate blood‐brain barrier (BBB) breakdown after ischemic stroke and lead to catastrophic hemorrhagic transformation (HT). Rosiglitazone(RSG), a widely used antidiabetic drug that activates peroxisome proliferator‐activated receptor‐γ (PPAR‐γ), has been shown to protect against cerebral ischemia through promoting poststroke microglial polarization toward the beneficial anti‐inflammatory phenotype. However, whether RSG can alleviate HT after delayed tPA treatment remains unknown. In this study, we sort to examine the role of RSG on tPA‐induced HT after stroke.

**Methods and results:**

We used the murine suture middle cerebral artery occlusion (MCAO) models of stroke followed by delayed administration of tPA (10 mg/kg, 2 hours after suture occlusion) to investigate the therapeutic potential of RSG against tPA‐induced HT. When RSG(6 mg/kg) was intraperitoneally administered 1 hour before MCAO in tPA‐treated MCAO mice, HT in the ischemic territory was significantly attenuated 1 day after stroke. In the tPA‐treated MCAO mice, we found RSG significantly mitigated BBB disruption and hemorrhage development compared to tPA‐alone‐treated stroke mice. Using flow cytometry and immunostaining, we confirmed that the expression of CD206 was significantly upregulated while the expression of iNOS was down‐regulated in microglia of the RSG‐treated mice. We further found that the expression of Arg‐1 was also upregulated in those tPA and RSG‐treated stroke mice and the protection against tPA‐induced HT and BBB disruption in these mice were abolished in the presence of PPAR‐γ antagonist GW9662 (4 mg/kg, 1 hour before dMCAO through intraperitoneal injection).

**Conclusions:**

RSG treatment protects against BBB damage and ameliorates HT in delayed tPA‐treated stroke mice by activating PPAR‐γ and favoring microglial polarization toward anti‐inflammatory phenotype.

## 1 INTRODUCTION

Tissue‐type plasminogen activator (tPA) is the only FDA‐approved drug therapy for acute ischemic stroke.^1^, ^2^, ^3^ Unfortunately, the administration of tPA may increase the risk of hemorrhagic transformation(HT), especially when delayed beyond 4.5 hours after the onset of ischemia,^4^, ^5^, ^6^ leading to poor clinical outcomes in stroke patients.^7^, ^8^, ^9^ Accumulating evidence suggests that HT is associated with disruption of blood‐brain barrier (BBB), which may occur early after stroke and largely limit the clinical use of tPA thrombolysis for stroke patients.^7^, ^10^, ^11^ Thus, there is an unmet need for developing an adjuvant agent that could protect the BBB integrity and extend the therapeutic window of tPA to benefit more stroke patients for safe thrombolysis and better functional recovery.^12^

Peroxisome proliferator‐activated receptor‐γ (PPAR‐γ), a ligand‐activated transcription factor belonging to the nuclear receptor superfamily, has been shown to orchestrate the microglia/macrophage phenotype switch from pro‐inflammatory to anti‐inflammatory phenotype, thus leading to inhibition of inflammation and tissue repair.^13^, ^14^, ^15^, ^16^ Rosiglitazone(RSG), a widely used antidiabetic drug with potent PPAR‐γ activating capacity, can protect against cerebral ischemia through its anti‐inflammatory and anti‐oxidant effect.^17^, ^18^, ^19^ However, it remains unknown whether RSG can be used as an adjuvant agent to protect the BBB integrity, especially during tPA thrombolysis after stroke. In this study, we sought to assess the effects of RSG on the protection of BBB integrity in tPA‐treated stroke mice and explore the underlying mechanism of RSG‐afforded protection against tPA‐induced HT after stroke.

## 2 MATERIALS AND METHODS

### 2.1 Murine model of transient focal ischemia

All animal experiments were approved by the Renji Hospital Institutional Animal Care and Use Committee and performed in accordance with the Institutional Guide for the Care and Use of Laboratory Animals. Focal cerebral ischemia was produced by intraluminal occlusion of the left middle cerebral artery (MCA) with a nylon monofilament suture as originally described with slight modifications.^4^, ^20^, ^21^ Male 2‐ to 3‐month‐old C57/B6 mice (25‐30 g each) were anesthetized with 1.5% isoflurane in a 30% O2/68.5% N2O mixture under spontaneous breathing. Rectal temperature was controlled at 37°C during and after surgery via a temperature‐regulated heating pad. The animals underwent left MCA occlusion (MCAO) for 2 hours and then were reperfused by withdrawing the suture. After recovering from anesthesia, the animals were maintained in an air‐conditioned room at 25°C.

### 2.2 Two‐dimensional laser speckle imaging techniques

Cortical blood flow was monitored using the laser speckle technique as described previously.^22^ Laser speckle perfusion images were obtained during middle cerebral artery occlusion and after reperfusion. Cerebral blood flow changes were recorded over time and expressed as a percentage of contralateral‐MCAO baselines.

### 2.3 Reperfusion with tPA and Rosiglitazone administration

At 2 hours after suture occlusion,the right femoral vein was cannulated for tPA administration. Recombinant human tPA (Actilyse; Boehringer Ingelheim) was intravenously administered at a dose of 10 mg/kg (10% as a bolus and 90% as a 30‐minute infusion) using a syringe infusion pump after reperfusion. RSG (Sigma‐Aldrich) was dissolved in dimethylsulfoxide and then further diluted in physiological saline (1:3 ratio), and was injected intraperitoneally (6 mg/kg) 1 hour before MCAO and at the time of tPA infusion. The animals that received administration of RSG or vehicle (25% dimethylsulfoxide) were assigned randomly to experimental groups. The optimal dose of the compound was administered as described before.^23^

### 2.4 Measurements of infarct volume

For microtubule‐associated protein 2 (MAP‐2) staining, animals were sacrificed and perfused transcardiacally with 0.9% normal saline and 4% paraformaldehyde in PBS. Free‐floating sections were prepared from the fixed and dehydrated brains and stained with MAP‐2 Abs (Santa Cruz Biotechnology). Infarct volume was determined with NIH Image J (1.52a) analysis by an observer “blinded” to the experimental group assignment.

### 2.5 Cresyl violet staining

Cresyl violet (CV) staining was performed to determine changes in intracerebral hemorrhage volume. Brain slices were mounted and left to dry for 24 hours at 4°C. Then, they were placed in a staining cage to dry at room temperature for 2 minutes. The slices were placed into a CV stain (0.1%, Solarbio) for 1.5 to 2 minutes. After that, they were placed in 100% alcohol solution concentrations to rehydrate for 5 minutes. Then, the slices placed in xylene for 5 minutes. Finally, slices were covered with Permount (Sinopharm chemical reagent Co., Ltd) and cover slipped. The images were captured on a laser scanning microscope (Olympus DP80, Olympus). The area of hemorrhage was quantified in the ipsilateral hemisphere in approximate sections per brain by a blinded investigator using NIH Image J (1.52a).

### 2.6 Immunofluorescence

Coronal sections were incubated with 10% normal donkey serum for 30 minutes at room temperature in PBS containing 0.1% Triton X‐100 followed by incubation with appropriate primary antibodies overnight at 4°C in the same buffer. The anti‐Iba‐1(1:500, Wako), anti‐Arg‐1 (1:500, Abcam), anti‐CD206(1:300, Abcam), and anti‐iNOS (1:300, BD Biosciences) primary antibodies were used. After primary antibody incubation, sections were washed four times at room temperature, followed by incubation with appropriate fluorescent‐labeled secondary antibodies (1:1000) for 1 hour at room temperature. DAPI was incubated for counterstaining of the nucleus. Sections were then washed with PBS and mounted with water‐based mounting medium containing antifading agents. All the confocal images were captured on a laser scanning confocal microscope (Olympus Fluoview FV3000, Olympus). The numbers of target immunopositive cells were quantified by a blinded investigator using NIH Image J (1.52a). Three randomly selected microscopic fields in the cortex on each section were analyzed for each brain by a blinded investigator. The immunopositive cell was presented as the mean percentage of cells per field (CD206 and iNOS measured at 40X magnification, Arg‐1 measured at 80X magnification).

### 2.7 Flow cytometry

We homogenized the hemisphere ipsilateral to the infarct using a Neural Tissue Dissociation Kit (MACS) by the gentle MACS Dissociator following the manufacturer's instructions (Miltenyi Biotec, Germany). The monocyte‐enriched population of cells was collected using a Percoll gradient.^24^ Cells were stained with APC‐Cy7‐CD45 (BD Biosciences), FITC‐CD11b (BD Biosciences), BV510‐CD86 (BD Biosciences), APC‐CD206 (Invitrogen), and the appropriate isotype controls (eBiosciences) following manufacturer's instructions, counted on the FACS Verse cell sorter (BD Biosciences) and analyzed using FlowJo software (TreeStar).

### 2.8 Statistical analysis

Sample sizes in different sets of experiments were calculated by power analyses based on pilot studies or the literature. Results were presented as mean ± SEM. The difference between means was assessed by the Student's t‐test for two‐group comparisons. One‐way ANOVA was used for multiple‐group comparisons, followed by post hoc Bonferroni/Dunn tests. The correlation analyses were performed using Pearson correlation analysis. *P* ≤ .05 was considered statistically significant.

## 3 RESULTS

### 3.1 RSG ameliorates delayed tPA‐induced HT in a suture model of stroke

**Figure 1:**
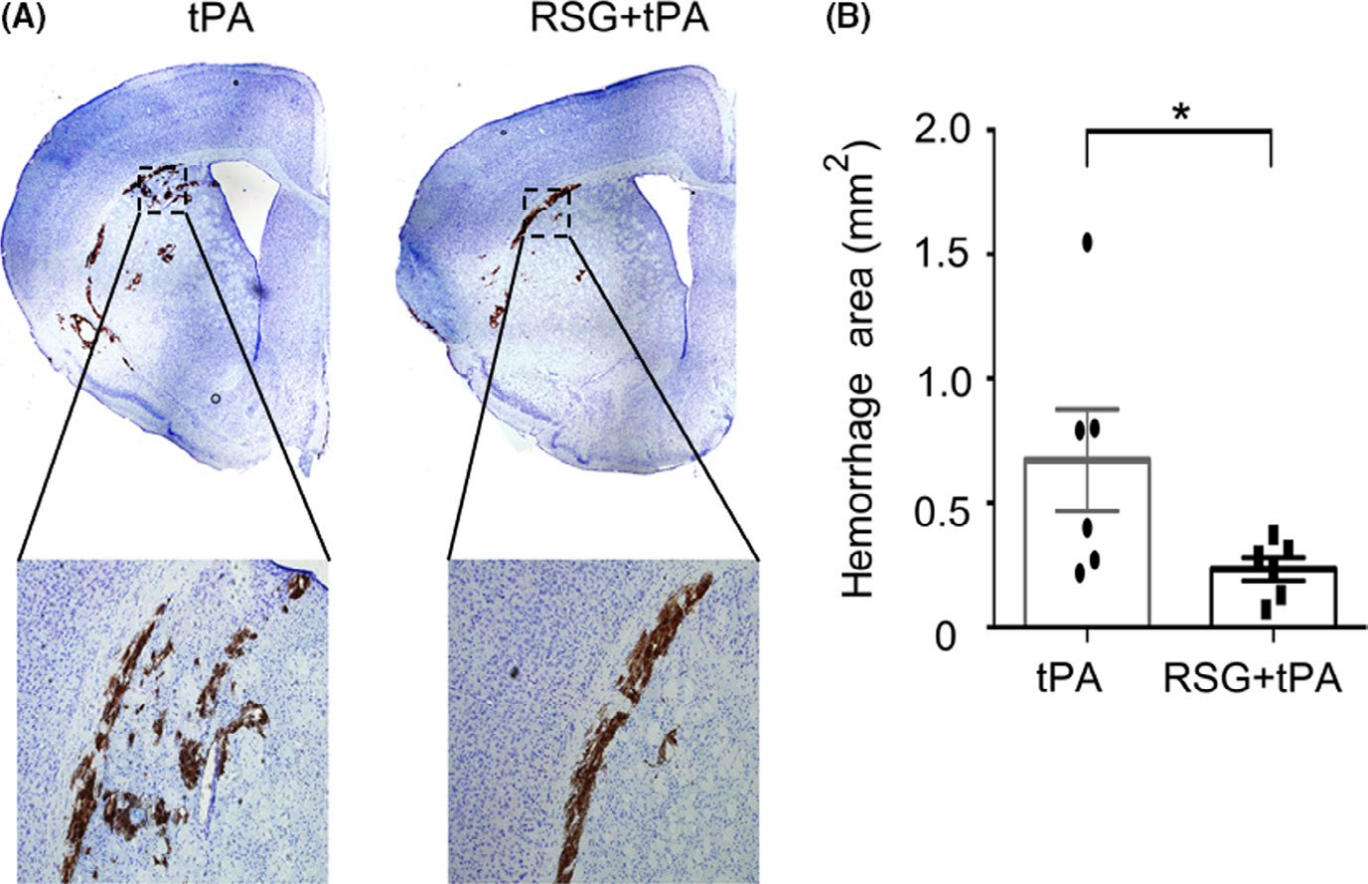
RSG significantly reduced hemorrhage area in tPA‐infused stroke mice. A, Representative images of intracerebral hemorrhage identified on cresyl violet (CV)‐stained coronal section. B, Quantification of hemorrhagic area 1 day after stroke in mice treated with tPA or RSG + tPA. Data are expressed as mean ± SEM. * *P* ≤ .05 vs tPA. RSG, rosiglitazone; tPA, tissue plasminogen activator

We first evaluated the effect of RSG on HT using the suture MCAO followed by tPA intravenous infusion (10 mg/kg）into the femoral vein in C57/BL6 mice. Massive hemorrhages were observed in the ischemic area at 1 day after ischemia/reperfusion in tPA‐treated mice; however, the hemorrhage was dramatically alleviated when RSG was administered 1 hour before MCAO in the tPA‐treated mice (Figure 1A‐B).

### 3.2 RSG reduces infarct volume in tPA‐infused stroke mice without affecting regional cerebral blood flow

**Figure 2:**
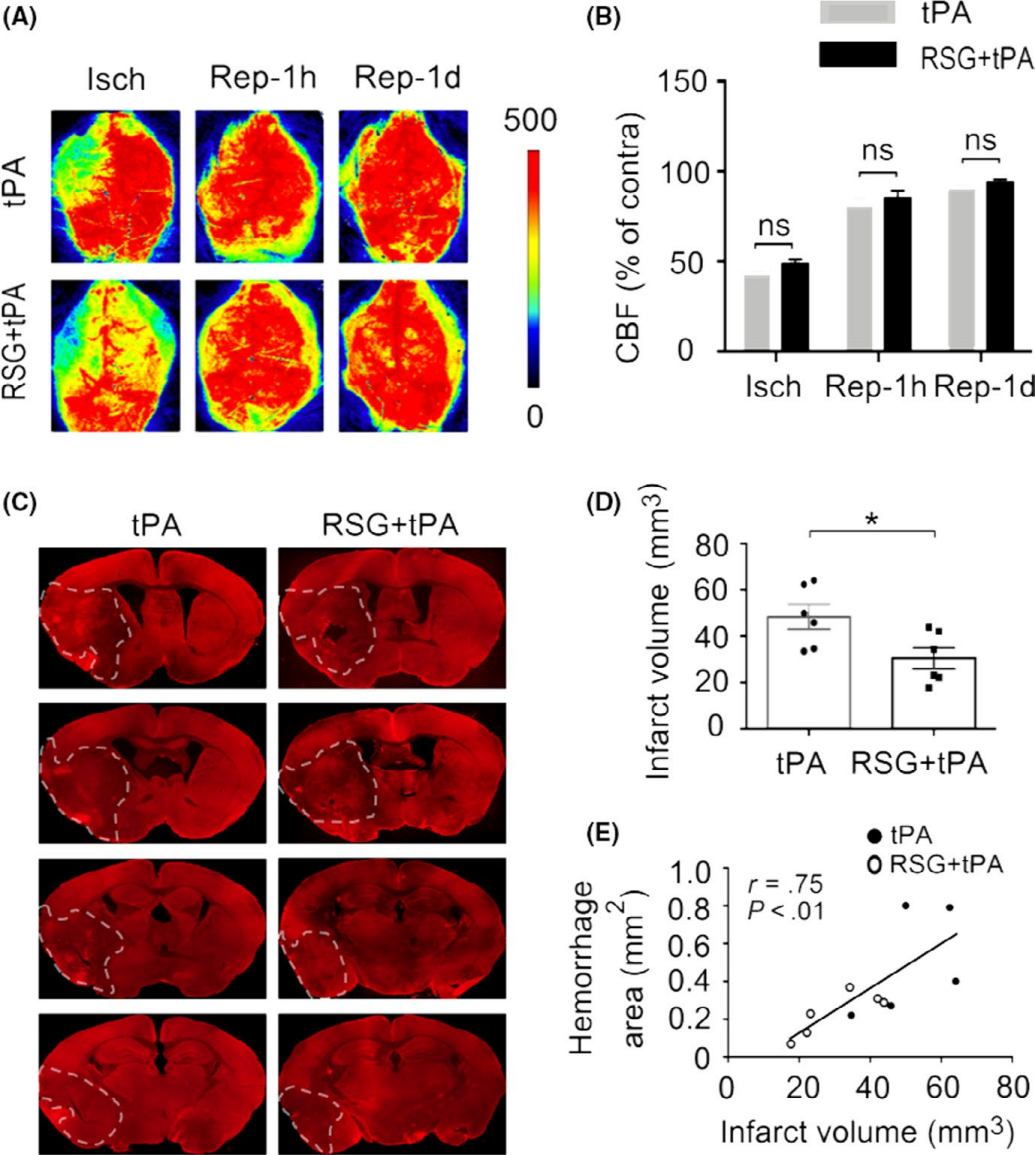
RSG reduces infarct volume in tPA‐infused stroke mice without affecting regional cerebral blood flow. A, Representative images of CBF during MCAO, 1h and 1d after reperfusion for each group. B, Quantification of CBF. Results are expressed as percent change from contralateral brain. n = 5‐6 per group. C, Representative MAP2 staining of brain infarct 1 day after stroke in mice treated with tPA or RSG + tPA. n = 6 per group. D, Quantification of infarct volume 1 day after stroke in mice treated with tPA or RSG + tPA. E, Correlation between infarct volume and hemorrhagic area in both experimental groups. Data are expressed as mean ± SEM. * *P* ≤ .05 vs tPA. RSG, rosiglitazone; tPA, tissue plasminogen activator; CBF, cerebral blood flow; MCAO, middle cerebral artery occlusion; MAP2, microtubule‐associated protein 2

We monitored regional cerebral blood flow during, at 1 hour and 1 day after MCAO by laser speckle 2D imaging (Figure 2A). There was no statistical difference in cerebral blood flow during and after MCAO between the two experimental groups, verifying that all animals were subjected to equivalent degree of ischemia and achieved equivalent extent of reperfusion (Figure 2B).

Next, we compared the infarct volume which was measured by MAP2 immunohistochemistry and found that RSG treatment significantly reduced the infarct volume in tPA‐treated stroke mice 1 day after MCAO (Figure 2C‐D). These results suggest that the combination of RSG with tPA treatment can reduce the ischemic brain infarct compared to tPA‐alone‐treated stroke mice. Next, we performed a correlation analysis and found that the infarct volume was positively correlated to the hemorrhage area in both experimental groups (Figure 2E).

### 3.3 RSG treatment blocks blood‐brain barrier disruption in tPA‐infused stroke mice

**Figure 3:**
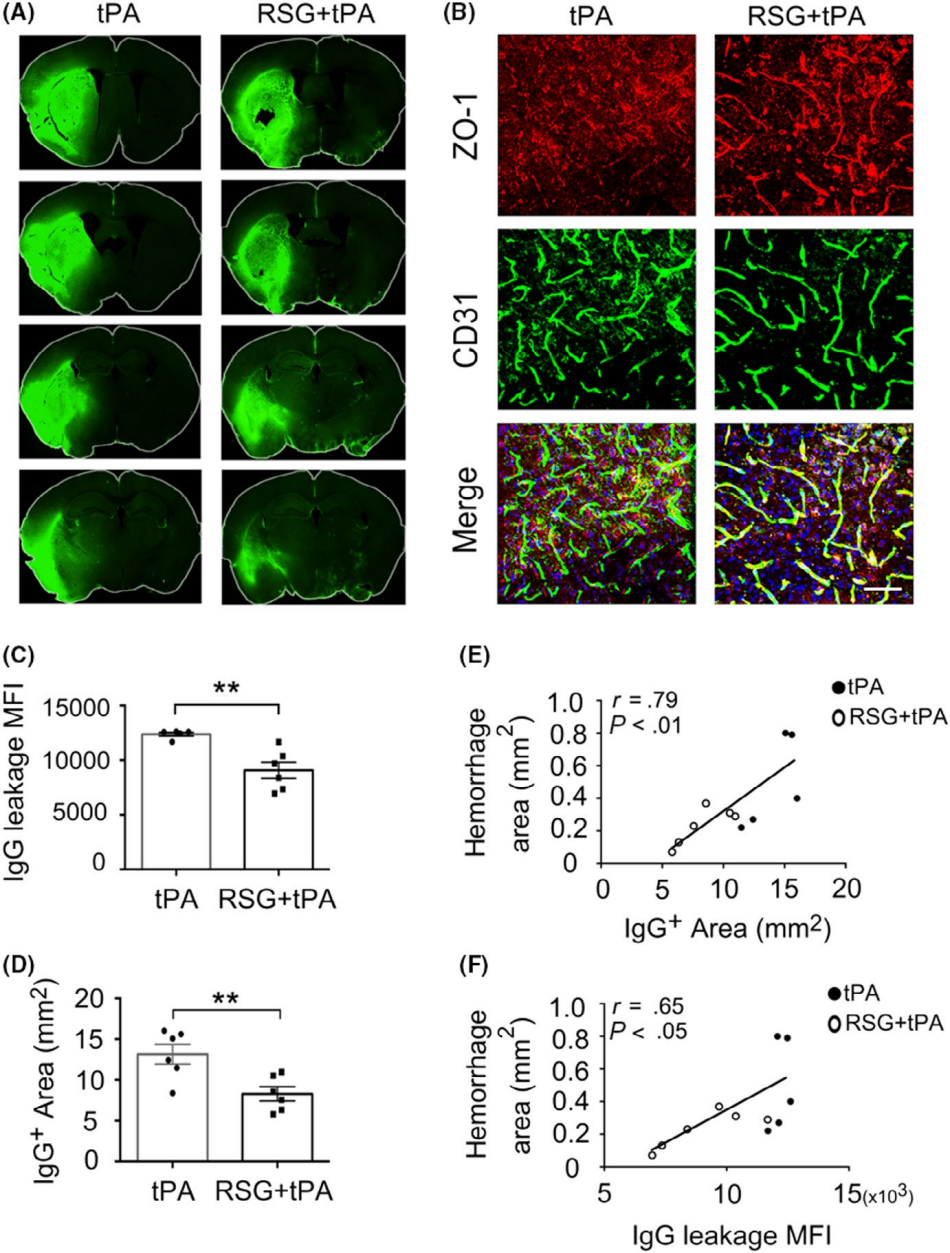
RSG attenuates blood‐brain barrier disruption in tPA‐infused stroke mice. A, BBB leakage was determined by immune‐staining of endogenous mouse IgG in the parenchyma. B, Representative Z‐stack confocal images of the tight junction protein ZO‐1 and endothelial cell marker CD31 in brain sections obtained 1 day after MCAO in mice treated with tPA or RSG + tPA. n = 5‐6 per group. Scale bar = 100μm. C, Quantification of MFI of IgG immunostaining 1 day after stroke in mice treated with tPA or RSG + tPA, n = 6 per group. D, Quantification of endogenous IgG positive area. n = 6 per group. E,F, Correlation between BBB disruption (IgG + area and IgG leakage MFI) and hemorrhagic area in both experimental groups. Data are expressed as mean ± SEM. ** *P* ≤ .01 vs tPA. RSG, rosiglitazone; tPA, tissue plasminogen activator; MCAO, middle cerebral artery occlusion; BBB, blood‐brain barrier; ZO‐1, Zonula occludens‐1; MFI, mean fluorescent intensity

Considering that BBB disruption is a critical factor that may contribute to the intracerebral hemorrhage,^25^, ^26^ we then explored the effect of RSG on the BBB integrity in the tPA‐treated stroke mice. By quantifying the extravasation of plasma‐derived IgG, we found that RSG treatment significantly reduced the BBB disruption in stroke mice with tPA thrombolysis (Figure 3A,C‐D). In addition, we measured the level of tight junction protein ZO‐1 using immunofluorescent staining and found that RSG treatment attenuated the disruption of ZO‐1 in tPA‐treated mice 1 day after MCAO (Figure 3B). Next, we did a correlation analysis and found that the BBB disruption (IgG^+^ area and IgG leakage MFI) was positively correlated with HT in both experimental groups (Figure 3E‐F).

### 3.4 RSG drives microglial/macrophage polarization in delayed tPA treatment after stroke

**Figure 4:**
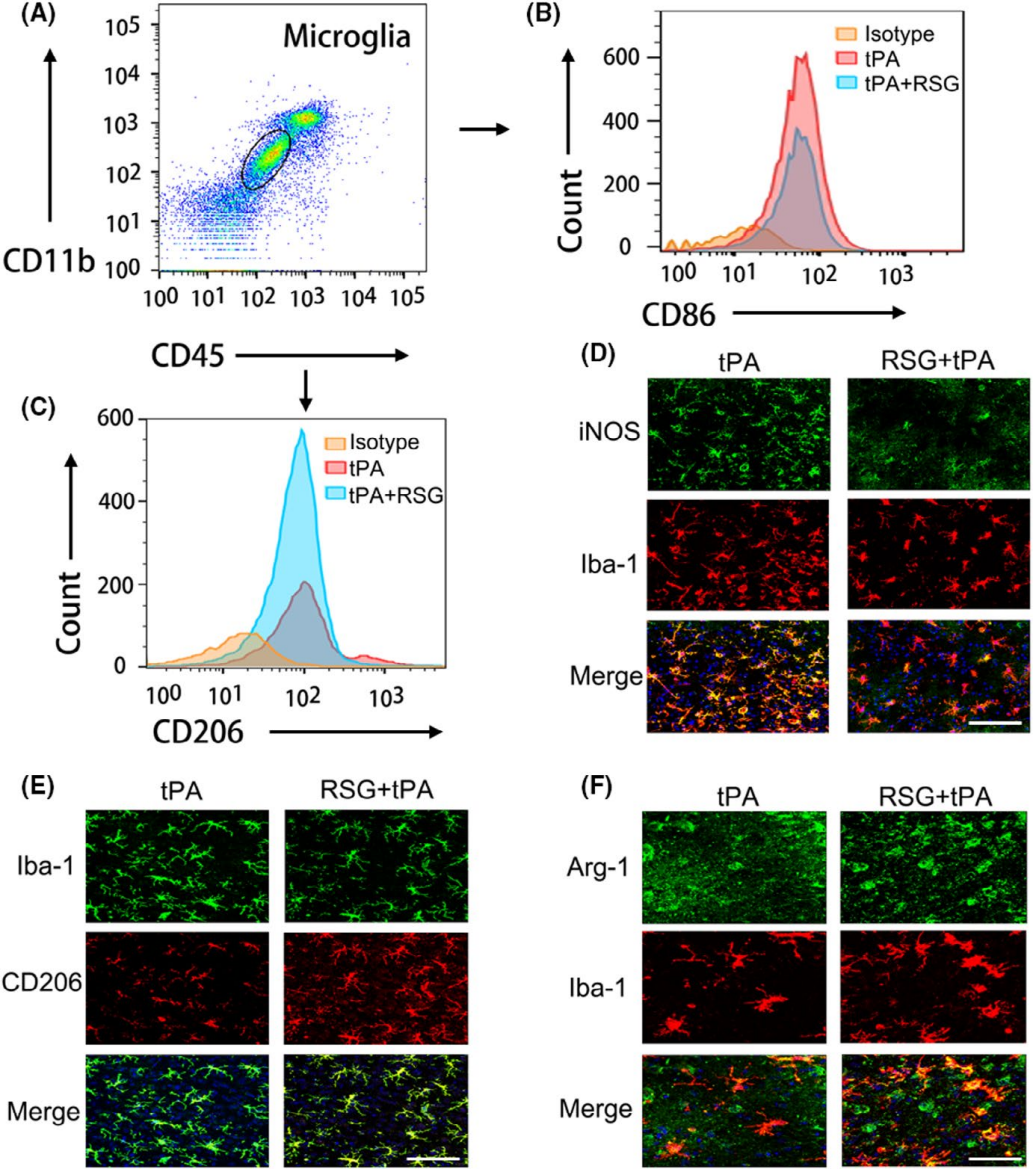
RSG drives anti‐inflammatory microglial polarization in delayed tPA treatment after stroke. A, Gating strategy for CD11b + CD45int cells in ischemic brain. B, Representative histogram of CD86 + microglia in ischemic brain 1 day in isotype, tPA, tPA + RSG groups (n = 6 per group). C, Representative histogram of CD206 + microglia in ischemic brain 1 day in isotype, tPA, tPA + RSG groups (n = 6 per group). D, Immunostaining of iNOS and Iba‐1 in brain sections 1 day after MCAO from mice treated with tPA or RSG + tPA. Scale bar = 100μm. E, Representative confocal images of CD206 and Iba‐1 double labeling in the brains obtained from tPA‐infused stroke mice with/without RSG. Scale bar = 100μm. F, Representative confocal images of Arg‐1 and Iba‐1 double immunostaining in the brains obtained from tPA‐infused stroke mice with/without RSG. Scale bar = 50μm. RSG, rosiglitazone; tPA, tissue plasminogen activator; MCAO, middle cerebral artery occlusion; iNOS, inducible nitric oxide synthase; Arg‐1, arginase 1; Iba‐1, ionized calcium‐binding adaptor molecule 1

**Figure 5:**
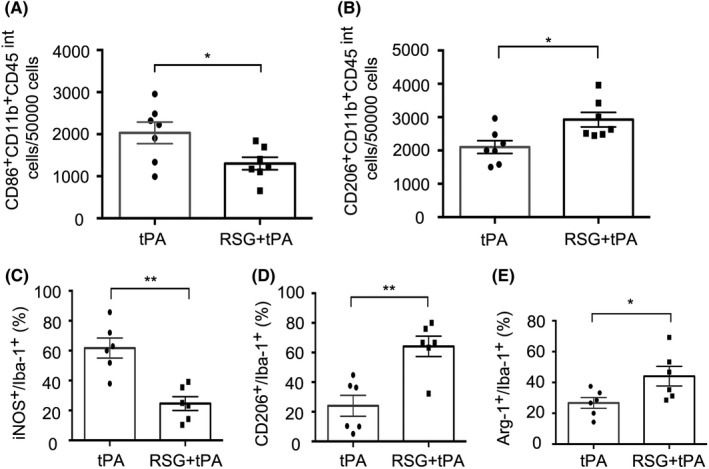
Quantification of microglia polarization in delayed tPA‐treated stroke mice with or without RSG. A, The cell number of CD86 + CD11b+CD45int microglia in ischemic brain (per 50 000 cells) was significantly decreased in RSG + tPA‐treated mice compared with tPA mice 1 day after MCAO. n = 7 per group. B, The cell number of CD206 + CD11b+ CD45int microglia in ischemic brain (per 50 000 cells) was significantly elevated in RSG + tPA‐treated mice compared with tPA mice 1 day after MCAO, n = 7 per group. C, Quantification of the percentage of iNOS+/Iba‐1 + cells in the ischemic brain, n = 6 per group. D, Quantification of the percentage of CD206+/Iba‐1 + cells in the ischemic brain, n = 6 per group. E, Quantification of the percentage of Arg‐1+/Iba‐1 + cells in the ischemic brain, n = 6 per group. Data are expressed as mean ± SEM. * *P* ≤ .05 vs tPA. RSG, rosiglitazone; tPA, tissue plasminogen activator; MCAO, middle cerebral artery occlusion; iNOS, inducible nitric oxide synthase; Arg‐1, arginase 1; Iba‐1, ionized calcium‐binding adaptor molecule 1

It has been suggested that RSG, a PPAR‐γ agonist, can drive microglial/macrophage polarization and phenotypic change,^27^ which is highly dynamic after ischemic injury.^10^ We next examined whether RSG treatment altered the function and phenotypic change of microglia/macrophage in the tPA‐treated stroke mice. Using flow cytometry, we found that the number of CD86^+^CD11b^+^CD45 ^int ^cells in the ischemic brain decreased significantly in RSG‐treated mice compared with those in the mice without RSG treatment 1 day after tPA thrombolysis (Figure 4A‐B and Figure 5A). Meanwhile, the number of CD206^+^CD11b^+^CD45^int^ cells in the ischemic brain increased significantly in RSG‐treated mice compared to those in the mice without RSG treatment 1 day after tPA thrombolysis (Figure 4A,C and Figure 5B). In order to further examine the effect of RSG on the polarization of microglia/ macrophages, we performed immunofluorescence staining and found that RSG treatment decreased the percentage of classically activated microglia/macrophages (iNOS^+^/Iba‐1^+^cells) (Figure 4D and Figure 5C)‚ while increased the percentage of alternatively activated microglia/macrophages (CD206^+^/Iba‐1^+^cells and Arg‐1^+^/Iba‐1^+^cells) (Figure 4E‐F and Figure 5D‐E). Collectively, these findings suggest that RSG promotes microglial polarization to the beneficial anti‐inflammatory phenotype after ischemia.

### 3.5 Anti‐inflammatory microglia polarization is associated with attenuated tPA‐induced HT and BBB disruption after stroke

**Figure 6:**
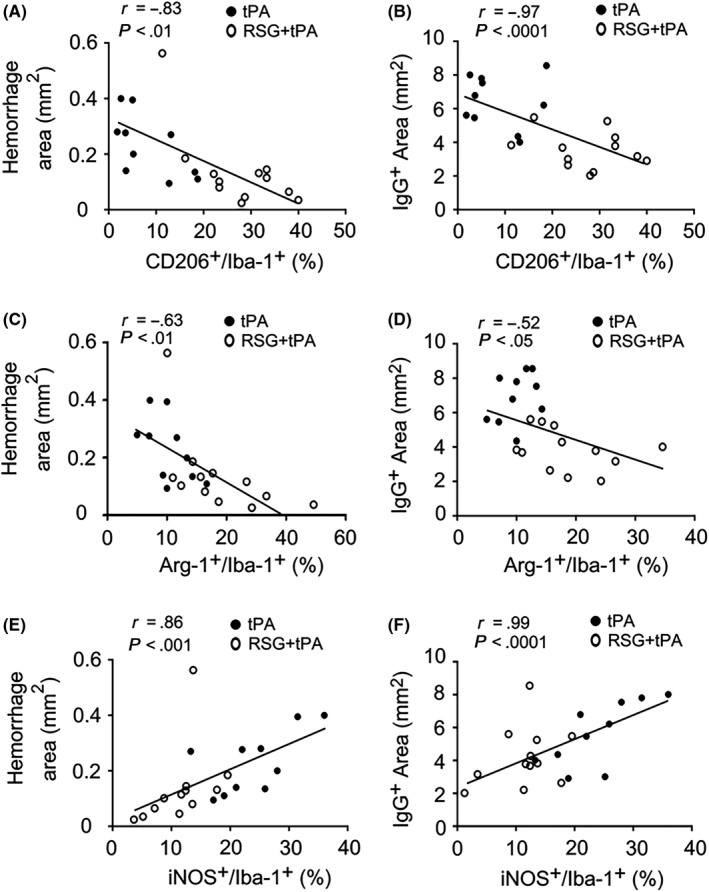
Anti‐inflammatory microglia polarization is associated with attenuated tPA‐induced HT and BBB disruption after stroke. A,B, Correlation analysis showed that the percentage of CD206+/Iba‐1 + cells was negatively correlated with hemorrhagic area/IgG + area. C,D, The percentage of Arg‐1+/Iba‐1 + cells was negatively correlated with hemorrhagic area/IgG + area. E,F, The hemorrhagic area and IgG + area was both correlated with the iNOS+/Iba‐1 + cells in both experimental groups. RSG, rosiglitazone; tPA, tissue plasminogen activator; HT, hemorrhagic transformation; MCAO, middle cerebral artery occlusion; iNOS, inducible nitric oxide synthase; Arg‐1, arginase 1; Iba‐1, ionized calcium‐binding adaptor molecule 1

Considering that RSG may promote anti‐inflammatory microglia polarization and meanwhile reduce the HT after tPA thrombolysis, we further analyzed the correlation between the anti‐inflammatory cellular markers, such as CD206 and Arg‐1 in Iba‐1^+^ cells in the brain with the hemorrhagic area and also the IgG^+^ area in the ischemic brain. We found that the percentage of CD206^+^/Iba‐1^+^ cells and Arg‐1^+^/Iba‐1^+^ cells were negatively correlated with hemorrhagic area and IgG^+^ area (Figure 6A‐D). We further analyzed the inflammatory marker iNOS with hemorrhagic area and IgG^+^ area in the ischemic brain, both of which were positively correlated with the percentage of iNOS^+^/Iba‐1^+^ cells (Figure 6E‐F). These results suggest that microglia polarization is closely associated with HT and BBB disruption after tPA thrombolysis for stroke.

### 3.6 PPAR‐γ antagonist GW9662 abolished the protection of RSG against HT and BBB disruption in tPA‐infused stroke mice

**Figure 7:**
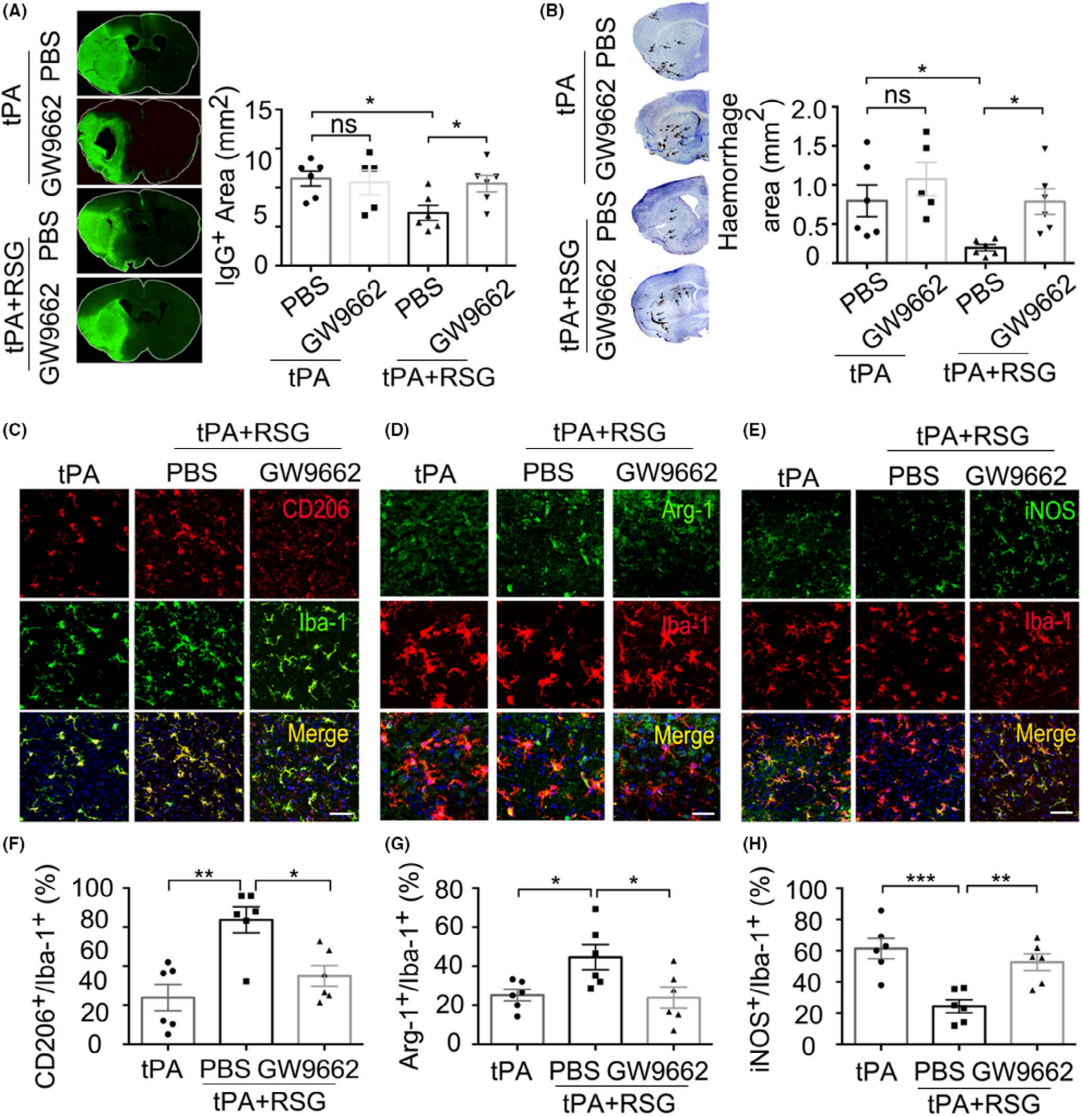
PPAR‐γ antagonist GW9662 abolished the protection of RSG against HT and BBB disruption in tPA‐infused stroke mice. A, Endogenous IgG extravasation into the parenchyma was visualized by staining sections with antibodies against mouse IgG molecules. B, Representative examples of images of intracerebral hemorrhage identified on cresyl violet (CV)‐stained coronal section. C, Representative confocal images of CD206 and Iba‐1 double immunostaining in the brains obtained from tPA‐infused stroke mice 1 day after MCAO treated with tPA, RSG and RSG + GW9662. Scale bar = 100μm. D, Representative confocal images of Arg‐1 and Iba‐1 double immunostaining in the brains obtained from tPA‐infused stroke mice 1 day after MCAO treated with tPA, RSG and RSG + GW9662. Scale bar = 50μm. E, iNOS and Iba‐1 double immunostaining 1 day after MCAO in mice treated with tPA, tPA + RSG and tPA + RSG+GW9662. Scale bar = 100μm. F, Quantification of the percentage of CD206+/Iba‐1 + cells in the brain, n = 6 per group. G, Quantification of the percentage of Arg‐1+/Iba‐1 + cells in the brain, n = 6 per group. H, Quantification of the percentage of iNOS+/Iba‐1 + cells in the brain, n = 6 per group. Data are expressed as mean ± SEM. * *P* ≤ .05, ** *P* ≤ .01 vs tPA. PPAR‐γ, Peroxisome proliferator‐activated receptor‐γ; RSG, rosiglitazone; tPA, tissue plasminogen activator; HT, hemorrhagic transformation; MCAO, middle cerebral artery occlusion; iNOS, inducible nitric oxide synthase; Arg‐1, arginase 1; Iba‐1, ionized calcium‐binding adaptor molecule 1

To further confirm that the protection of RSG against HT and BBB disruption in tPA‐treated stroke mice was exerted through activating PPAR‐γ, we administered the PPAR‐γ antagonist GW9662 (4 mg/kg) along with RSG 1 hour before MCAO through intraperitoneal injection. We found that GW9662 abolished the protection of RSG against HT and BBB disruption in tPA‐treated stroke mice 1 day after stroke (Figure 7A‐B). In addition, GW9662 treatment alone did not significantly alter the BBB disruption after stroke (Figure 7A‐B). We further analyzed the microglia phenotype and found that the microglia polarization was also reversed by GW9662 treatment together with RSG 1 day after stroke (Figure 7C‐G). These results suggest that RSG exerted its protection against HT and BBB disruption via activating PPAR‐γ in tPA‐treated stroke mice.

## 4 DISCUSSION

The present study investigated the effect of RSG treatment 1 hour prior to postischemic reperfusion on HT and BBB disruption in stroke mice with tPA thrombolysis. We found that RSG reduced acute brain infarction and attenuated HT and BBB disruption in tPA‐treated mice 1 day after stroke. These protective effects of RSG are closely associated with microglia polarization toward the anti‐inflammatory phenotype, which may be mediated by activating PPAR‐γ. The current finding may pave the way to the development of adjuvant treatment with tPA thrombolysis after stroke in the pursuit of extending the therapeutic window of tPA.

Early reestablishment of cerebral blood flow is crucial for the functional recovery of acute ischemic stroke patients.^28^, ^29^ The latest guideline of acute stroke treatment still recommends tPA as the first‐line of effective thrombolytic treatment to achieve early reperfusion of the ischemic brain.^2^, ^3^, ^30^ However, the current therapeutic window of 4.5 hours after the onset of stroke remarkably limits the clinical use of tPA thrombolysis.^31^, ^32^, ^33^ BBB disruption is a fundamental pathological feature of intracerebral HT induced by tPA thrombolysis.^34^, ^35^ Loss of BBB integrity results in increased vascular permeability and is associated with reduced cerebral blood flow and impaired hemodynamic responses.^35^, ^36^ Thus, finding a novel strategy to preserve the BBB integrity during tPA thrombolysis is crucial to reduce the risk of intracerebral hemorrhage.

RSG is a potent thiazolidinedione which activates the PPAR‐γ. It has been shown to exert neuroprotective effects against cerebral ischemic stroke both in animal models and stroke patients.^37^ Administration of RSG at the dose of 6 mg/kg 1 hour prior to MCAO could significantly reduce brain infarct at 48 hours after stroke.^23^ In order to explore the effect of RSG on the tPA‐induced HT after stroke, we injected RSG at the above‐mentioned dose and found that RSG significantly reduced hemorrhagic area, IgG extravasation, and tight junction protein degradation.

As a widely used antidiabetic drug which works as an insulin sensitizer by binding to the PPAR‐γ in fat cells, RSG may also serve as a master gatekeeper in CNS injury and repair.^37^, ^38^, ^39^ It is emerging that the PPAR‐γ is constitutively expressed in microglia and activating PPAR‐γ in microglia may regulate the phenotypic change of microglia.^40^, ^41^, ^42^, ^43^ PPAR‐γ activation could favor a reprogramming process of microglia toward a beneficial phenotype, with anti‐inflammatory and tissue‐repair properties.^10^, ^11^, ^12^, ^13^, ^44^ Interestingly, previous studies have shown that 1 day after ischemia, the microglia/macrophages adopt an anti‐inflammatory phenotype that progressively evolves toward a neurotoxic phenotype at day 7 poststroke.^45^ Depletion of monocytes/macrophages increases the ischemic lesion; the ratio of inflammatory/anti‐inflammatory macrophages polarization ratio increases early in a mouse model of stroke.^46^ In order to address whether the protection of RSG against tPA‐induced HT is mediated by its PPAR‐γ activating effect, we used the PPAR‐γ antagonist GW9662 in combination with RSG in the tPA‐treated stroke mice. We found that the therapeutic effect of RSG against tPA‐induced HT and BBB disruption was abolished in the presence of GW9662, suggesting a critical role of PPAR‐γ activation in the RSG‐afforded protection in tPA‐induced HT after stroke.

In conclusion, we find that RSG treatment attenuates HT and BBB disruption in tPA‐treated stroke mice. The RSG‐afforded protective effects on HT and BBB damage are correlated with the microglial polarization toward the anti‐inflammatory phenotype and the protection of RSG may be mediated by PPAR‐γ activation.

## CONFLICT OF INTEREST

The authors declare no conflict of interest.
